# The impact of COVID-19 infection on the live birth rate in fresh embryo transfer cycles

**DOI:** 10.3389/fendo.2026.1764229

**Published:** 2026-04-17

**Authors:** Dan Sun, Lu Wang, Ying Su, Ni Jin, Juan Zhou, Ling Li, Yan Zhang, Xiaohong Wang, Huishou Zhao, Shuqiang Chen, Ying Ju

**Affiliations:** 1 Department of Obstetrics and Gynecology, Reproductive Medicine Center, Tang Du Hospital, Fourth Military Medical University, Xi’an, China; 2 Department of Cardiology, Xijing Hospital, Fourth Military Medical University, Xi’an, China

**Keywords:** COVID-19, fresh embryo transfer, *in vitro* fertilization, live birth rate, neonatal outcomes

## Abstract

**Background and aims:**

It is important to clarify the impact of COVID-19 on ART outcomes and to develop evidence-based guidelines for deciding whether to proceed with or cancel *in vitro* fertility procedures in infected patients. This study aims to clarify the specific impacts of COVID-19 infection on the live birth rates (LBR) and fetal outcomes in patients undergoing fresh embryo transfer (fET) cycles.

**Methods:**

This retrospective study analyzed 1,025 fresh embryo transfer cycles from January 2021 to January 2023. We compared pregnancy and neonatal outcomes between the non-infected group (n=762) and the COVID-19-infected group (n=263). Additionally, we stratified the infected group into subgroups by two criteria: time of infection, with or without fever and partner infection status, and further compared pregnancy outcomes of these subgroups. Univariate and multivariate logistic regression analyses were performed to assess differences in pregnancy and neonatal outcomes between non-infected and COVID-19-infected individuals.

**Results:**

The newborn’s gender, birth height and birth weight were comparable between the infected and uninfected groups. However, the COVID-19 infection group exhibited a lower LBR with an adjusted odds ratio (OR) of 0.655 (95% confidence interval (CI: 0.483 to 0.887; *P =* 0.006) and a higher mid-to-late miscarriage rates with an adjusted OR of 7.929 (95% CI: 2.651 to 23.714; *P <* 0.001) compared with the non-infections group. Stratified analysis showed that infections occurring between 28 and 84 days prior to oocyte retrieval accompanied by fever of ≥ 38.5°C resulted in a lower LBR (OR: 0.467, 95% CI: 0.290 to 0.752; *P =* 0.002 < 0.025) after Bonferroni’s correction. Additionally, the dual-partner infection group demonstrated a significantly reduced LBR (OR: 0.591, 95% CI: 0.421-0.830; *P* = 0.002 < 0.025) compared to uninfected controls.

**Conclusion:**

COVID-19 infection was associated with a lower LBR in fET cycles, especially when: infection occurred 28–84 days before oocyte retrieval with fever ≥ 38.5°C, or dual-partner were infected. Subsequent multicenter studies enrolling a significantly larger cohort of infected women or couples are essential to validate this finding.

## 1 Introduction

COVID-19, caused by severe acute respiratory syndrome coronavirus 2 (SARS-CoV-2), is an infectious respiratory disease that has triggered a global pandemic since 2020 (1). Following the easing of pandemic restrictions globally, a substantial rise in COVID-19 infections among couples undergoing IVF treatment cycles has been observed. Recently, the long-term multi-system syndrome and health crisis brought by COVID-19 cases raises the concerns about its impact on IVF reproductive outcomes (2).

SARS-CoV-2 enters target host cells via the cellular receptor angiotensin-converting enzyme 2 (ACE2) (3). Notably, ACE2 is highly expressed in human reproduction system, such as ovaries and testis (4, 5). Studies also shown that ACE2 is expressed in oocytes (6), granulosa cells and pre‐ovulation follicles, blastocysts (4, 7). Interestingly, ACE2 mRNA levels of uterus epithelial cells and stromal cells are dramatically enhanced in the secretory phase, compared to the proliferative phase (3, 8). During pregnancy, ACE2 is found to be widely expressed in the human placenta (9). In addition, SARS-CoV-2 virus has been identified in semen (10), follicular fluid (11) and placental samples (12), which suggest that COVID-19 infection may affect pregnancy outcomes.

However, the effect of COVID-19 infection upon IVF clinical outcomes are inconsistent. In fresh cycles, current studies indicate that patients with a history of COVID-19 infection exhibit no statistically significant differences in key reproductive outcomes—including oocyte yield, oocyte maturation rates, fertilization rates, number of vitrified embryos, or clinical pregnancy rates—when compared to non-infected controls (13, 14). While some studies found that COVID-19 patients had a significantly lower embryo morphokinetics and a higher miscarriage rate (15, 16). Thus, the key recommendations from the American Society for Reproductive Medicine (ASRM) were to suspend initiation of new treatment cycles, including ovulation induction, intrauterine inseminations and *in vitro* fertilization. The Society of Gynecologic Surgeons and the Society for Maternal-Fetal Medicine also endorses the suspension of medically indicated procedures except in cases where a delay in treatment would negatively affect the health and safety of the patient. These highlight the importance of clarifying COVID-19’s impact on ART outcomes, and developing evidence-based guidelines for deciding whether to proceed with IVF procedures or cancel them in infected patients.

In this study, we focus on the impact of COVID-19 on LBR and neonatal outcomes after fET. We further conducted stratified analyses evaluating COVID-19’s impact on LBR across three clinical dimensions (1): the interval between COVID-19 infection and oocyte retrieval (2), infection severity, particularly fever magnitude (3). single infected or dual infected. Our work will be helpful to develop an evidence-based framework for optimizing assisted reproductive technology (ART) management in patients infected with COVID-19 infection.

## 2 Methods

### 2.1 Study design and participants

This retrospective cohort study included all patients who underwent fresh IVF treatment cycles between 1 January 2021 and 31 January 2023 at the Center of Assisted Reproduction, Tangdu Hospital, Fourth Military Medical University, China. Exclusion criteria were as follows (1): use of donor semen (2); incomplete SARS-CoV-2 infection documentation (3); uterine malformation or endometrial factors (4); history of cervical surgery or cervical incompetence-related pregnancy loss/preterm birth; and (5) undergone more than three gonadotrophin cycles aiming to eliminate bias in treatment outcomes caused by individual factors such as recurrent implantation failure and oocyte abnormalities.

**Figure 1:**
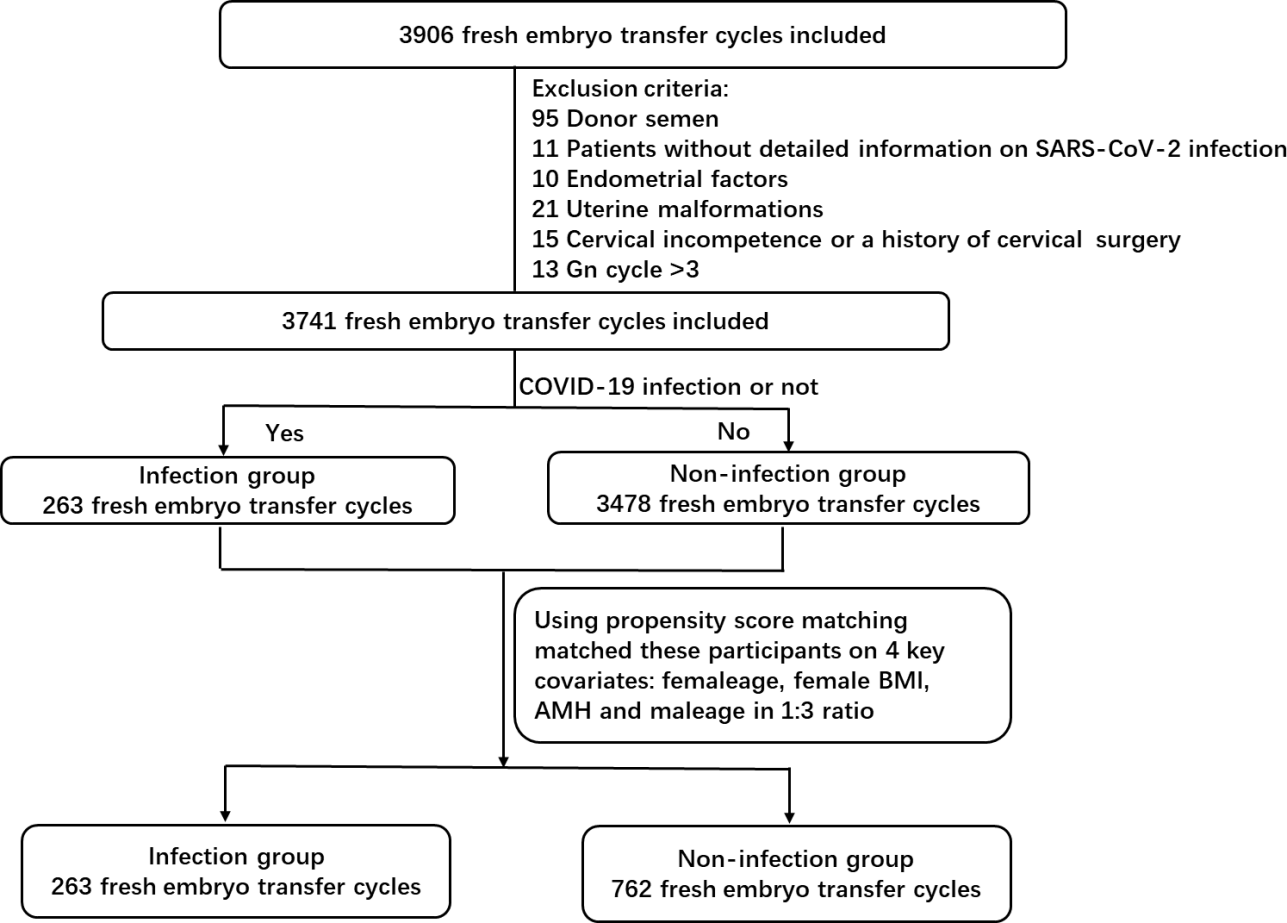
Flowchart of patient inclusion. Gn: gonadotrophin; BMI, body mass index; AMH, anti-Müllerian hormone.

In this study, all participants were enrolled in the IVF cycle only after testing negative for COVID-19, besides they underwent COVID-19 testing every other day during the study period using SARS-CoV-2 nucleic acid or antigen assays. The study population was stratified into two groups based on the female participants’ COVID-19 infection status: infection group (females with individual infection or couples with both partners infected) and non-infection group (both partners uninfected). Propensity score matching (PSM) was employed to balance key covariates influencing outcomes: female age, body mass index (BMI), male age, and anti-Müllerian hormone (AMH) levels. This approach successfully equilibrated both groups for these four variables. The participant selection flowchart was presented in **Figure 1**. Ultimately, 1,025 treatment cycles were analyzed (non-infection group: 762 cycles; infection group: 263 cycles).

### 2.2 IVF/ICSI protocols

Ovarian stimulation was performed using the GnRH agonist long protocol, GnRH antagonist protocol, and other regimens including the minimal stimulation protocol, short GnRH agonist protocol, and ultra-short GnRH agonist protocol. Follicular development was monitored via transvaginal ultrasound. When at least three follicles reached ≥17 mm in diameter, 250 µg recombinant human chorionic gonadotropin (rhCG) was administered subcutaneously for ovulation triggering. Oocyte retrieval was performed 36–38 hours post-trigger (17, 18). Fertilization assessment (conventional IVF or intracytoplasmic sperm injection (ICSI)) was conducted 12–17 hours post-insemination (18). Cleavage-stage embryos were transferred on day 3 post-retrieval, while blastocysts were transferred on day 5. Luteal phase support comprised daily vaginal progesterone supplemented with optional oral dydrogesterone (20 mg) when clinically indicated (19). Biochemical pregnancy was confirmed by serum β-hCG > 5 IU/L measured 15 days after cleavage-stage transfer or 13 days after blastocyst transfer (18). Throughout the study period, clinical protocols, laboratory conditions, culture media, and embryo transfer techniques were similar.

### 2.3 Study variables

Demographic and cycle characteristics data were collected, encompassing the ages of both partners, maternal body mass index (BMI), duration of infertility, AMH levels, infertility type and etiology (containing female factors, male factors, mixed factors (both female and male factors) and unexplained factors), Semen parameters (volume, density, progressive motility, morphology), gonadotrophin (Gn) dosage and administration duration and ovarian stimulation protocols. Additionally, the number of retrieved oocytes, fertility treatment methods, and endometrial thickness were recorded. Information on the embryo transfer stage, the quantity of retrieved metaphase II (MII) oocytes, and the normal fertilization rate (calculated as the ratio of normally fertilized oocytes to the total number of retrieved oocytes) was also documented.

### 2.4 Definitions and measures of outcomes

Pregnancy outcome indicators included embryo implantation rates, clinical pregnancy rates, biochemical pregnancy rates, LBR, early miscarriage rates, mid-to-late miscarriage rates, ectopic pregnancy rates, premature birth rates, ovarian hyperstimulation syndrome (OHSS) incidence, and the overall complications associated with pregnancy. The embryo implantation rate is defined as the percentage of transferred embryos that successfully implant in the uterus. The clinical pregnancy rate is defined as the percentage of IVF cycles confirmed by ultrasound. The biochemical pregnancy rate refers to the percentage of IVF cycles in which a pregnancy is initially detected through a positive beta-human chorionic gonadotropin (β-hCG) blood test but where no gestational sac or fetal heartbeat is confirmed via ultrasound.

The LBR was defined as the percentage of IVF cycles resulting in the birth of a living infant. Early miscarriage (occurring before 12 weeks), mid-to-late miscarriage (occurring between 12–28 weeks) (20), and premature birth (occurring between 28–36 weeks) were classified based on gestational age. Because premature birth rates are ten times higher in twin pregnancies than in singleton pregnancies (21), The metric was recorded exclusively for singleton pregnancies. The ectopic pregnancy rate denotes the percentage of IVF pregnancies where an embryo implants outside the uterine cavity.

Neonatal outcome indicators included newborn gender, height, and weight. The gender of all live-born infants was recorded, while height and weight measurements were taken exclusively for singletons. According to World Health Organization (WHO) definitions, normal birth weight is classified as ranging from 2500 to 4000 g; low birth weight (LBW) is defined as less than 2500 g; very low birth weight is categorized as less than 1500 g; and macrosomia refers to a birth weight greater than 4000 g.

### 2.5 Statistical analysis

For continuous variables, normality of distribution was initially assessed using the Shapiro-Wilk test. Normally distributed variables are presented as mean ± standard deviation and compared between groups using Student’s t-tests. Non-normally distributed variables are summarized as median (Q1, Q3) and compared using the Kruskal-Wallis test. Categorical variables are expressed as counts (percentages) with differences assessed using the Chi-square test or Fisher’s exact test. Univariate and multivariate logistic regression analyses were performed to identify preliminary associations, adjusting for confounding factors closely related to LBR. Results are presented as odds ratios (OR) with 95% confidence intervals.

Additionally, subgroup analyses were performed based on the time of infection (less than 28 days before retrieval; more than 28 days but less than 84 days before retrieval) and partner infection status (only the female infected; both partners infected). Furthermore, participants infected 28–84 days before retrieval were stratified by peak body temperature during fever: < 37.2°C and < 38.5°C or ≥ 38.5°C. Pairwise comparisons between groups were adjusted by the Bonferroni method for multiple testing, with the adjusted significance level (α) calculated as 0.05/2 = 0.025. Statistical significance was defined as a two-sided *P*-value < 0.05 or *P*-value < 0.025. All analyses were conducted using R (version 4.4.2) and SPSS (version 27).

## 3 Results

### 3.1 Study population

A total of 3,741 fresh cycles were screened during the study period. Through propensity score matching (1:3 ratio) based on female age, body mass index, male age, and AMH levels, 1025 cycles were included in this study, of which 263 SARS-CoV-2-positive partners were assigned to the COVID-19 infection group and 762 SARS-CoV-2-negative partners to the non-infection group. **Figure 1** illustrates a flow chart of the study population.

### 3.2 Baseline characteristics, cycle parameters and cycle outcomes

The baseline characteristics, cycle parameters and cycle outcomes of both groups are presented in **Table 1**. No statistically significant differences were observed in maternal age, paternal age, maternal BMI, infertility duration, AMH level, infertility type, semen volume, sperm progressive motility, sperm abnormality rates, Gn duration, the number of oocytes retrieved, fertilization method, the number of MII oocytes, the number of embryos transferred, or endometrial thickness. Compared to the non-infection group, males in the infection group exhibited significantly lower sperm concentration (55 (35,79) vs. 51(30,75), *P* < 0.001) and a higher proportion of mixed-factor infertility (2.0% vs. 14.1%, *P* < 0.001). Regarding cycle parameters, the infection group showed reduced utilization of the GnRH-a long protocol (58.1% vs. 51.0%, *P* < 0.001), increased adoption of alternative protocols (2.9% vs. 8.4%, *P* < 0.001), higher Gn dosage (1950 (1400,2896.88) IU vs. 2275(1575,3150) IU, *P* < 0.001) and lower normal fertilization rate (80 (66.67, 90.91)% vs. 81.82 (69.23, 100)%, *P* = 0.014), and these differential confounding factors have all been adjusted for in the multivariate logistic regression analysis.

**Table 1:** Comparison of baseline characteristics and cycle parameters between non-infection group and infection group.

Characteristics	Non-infection group	Infection group	*P*-value
Cycles (n)	762	263	
Baseline characteristics			
Maternal age (year), mean ± SD	30.24 ± 3.59	30.74 ± 3.85	0.061
Paternal age (year), mean ± SD	30.07 ± 2.20	30.12 ± 0.89	0.628
Maternal BMI (kg/m2), median (Q1,Q3)	22.72 (20.65, 24.97)	20.38 (22.27, 24.84)	0.238
Infertility duration (year), median (Q1,Q3)	3 (1, 4)	3 (1, 5)	0.071
AMH (ng/ml), median (Q1,Q3)	1.94 (1.11, 3.10)	1.79 (0.97, 3.03)	0.189
Type of infertility, n (%)			0.918
Primary	420 (55.1)	144 (54.8)	
Secondary	342 (44.9)	119 (45.2)	
Infertility cause, n (%)			<0.001*
Female	568 (74.5)	184 (70.0)	
Male	142 (18.6)	35 (13.3)	
Mixed	15 (2.0)	37 (14.1)	
Unexplained	37 (4.9)	7 (2.7)	
Semen volume, median (Q1,Q3)	3.0 (2, 3.5)	3 (2, 3.5)	0.937
Sperm concentration, median (Q1,Q3)	55 (35, 79)	51 (30, 75)	0.028*
Semen progressive mobility, median (Q1,Q3)	35 (27, 43)	36 (25, 50)	0.071
Sperm abnormality rate (%), median (Q1,Q3)	95.5 (94, 96)	95 (94, 96)	0.692
Cycle parameters			
Ovarian stimulation protocols, n (%)			<0.001*
GnRH-a long protocol	443 (58.1)	134 (51.0)	
Antagonist protocol	297 (39.0)	107 (40.7)	
Other protocols	22 (2.9)	22 (8.4)	
Gn duration (day), median (Q1,Q3)	11 (10, 13)	11 (10, 13)	0.756
Gn dosage (IU), median (Q1,Q3)	1950 (1400, 2896.88)	2275 (1575, 3150)	<0.001*
Fertility method, n(%)			0.316
IVF	504 (66.1)	161 (61.2)	
ICSI	215 (28.2)	108 (33.1)	
IVF+ICSI	43 (5.6)	9 (5.7)	
Endometrial thickness (mm), median (Q1,Q3)	11 (10, 12)	11 (10, 12)	0.116
Number of embryos transferred, n (%)			0.249
1	438 (57.5)	140 (53.2)	
2	324 (42.5)	123 (46.8)	
Cycle outcomes			
Number of oocytes retrieved, median (Q1,Q3)	9 (6, 12)	9 (6, 13)	0.561
Retrieved MII oocytes, median (Q1,Q3)	8 (5, 10)	7 (5, 10)	0.132
Normal fertilization rate (%), median (Q1,Q3)	80 (66.67, 90.91)	81.82 (69.23, 100)	0.014*

Data are shown as mean ± standard deviation (SD), median (Q1, Q3) or number (percentage).

OHSS, ovarian hyperstimulation syndrome; ICSI, intracytoplasmic sperm injection; MII, metaphase II.

*Statistically significant with *P* < 0.05.

### 3.3 Pregnancy outcomes

Pregnancy outcomes of the non-infection group and infection group are presented in **Table 2**. There were no significant differences in the rates of OHSS, implantation, clinical pregnancy, biochemical pregnancy, early miscarriage, ectopic pregnancy, or premature birth. Notably, the infection group exhibited a significantly lower LBR (46.9% vs. 36.1%, *P* = 0.003) and a higher mid-to-late miscarriage rate (1.5% vs. 9.4%, *P* < 0.001), compared with the non-infection group (**Supplementary Table 1**). After adjusting for potential confounders, multivariate logistic regression demonstrated that infection was significantly negatively associated with LBR (adjusted OR: 0.655, 95% CI: 0.483–0.887), and strongly positively associated with mid-to-late miscarriage rate (adjusted OR: 7.929, 95% CI: 2.651–23.714) (**Table 2**).

**Table 2:** Univariate and multivariate logistic regression analysis of pregnancy outcomes.

Pregnancy outcomes	Crude OR	95% CI	*P*1-value	Adjusted OR	95% CI	*P*2-value
Noninfection group	ref			ref		
Infection group						
Implantation	0.799	0.631,1.013	0.064	0.809	0.631,1.037	0.094
Clinical pregnancy	0.664	0.400,1.103	0.114	0.681	0.399,1.160	0.157
Biochemical pregnancy	0.821	0.409,1.647	0.579	0.811	0.393,1.675	0.572
Early miscarriage	1.625	0.765,3.451	0.206	1.558	0.705,3.445	0.273
Mid-to-late miscarriage	6.835	2.501,18.612	<0.001	7.929	2.651,23.714	<0.001*
Ectopic pregnancy	1.049	0.332,3.311	0.935	0.899	0.257,3.145	0.868
LBR	0.642	0.480,0.857	0.003	0.655	0.483,0.887	0.006*
Premature birth	0.845	0.310,2.303	0.742	0.895	0.322,2.489	0.831

Analyses were adjusted for Gn dose, sperm concentration, normal fertilization rate, ovarian stimulation.

protocols and infertility factor. OR, Odds ratio; CI, confidence interval.

*Statistically significant with p<0.05.

### 3.4 Neonatal outcomes analysis

Neonatal outcomes are presented in **Table 3** and **Supplementary Table 2**. To mitigate confounding effects of multiple gestations, twin pregnancies were excluded from birth weight analysis in both study groups. After adjustment for potential confounders, no significant differences were observed in sex distribution, birth height, very low birthweight (< 1500 g), low birthweight (< 2500 g), or macrosomia (≥ 4000 g) between the non-infection and infection groups.

**Table 3:** Univariate and multivariate logistic regression analysis of neonatal outcomes.

Neonatal outcomes	Crude OR	95% CI	*P*1-value	Adjusted OR	95% CI	*P*2-value
non-infection group	Ref			Ref		
Infection subgroup						
Gender of newborn	1.003	0.654,1.540	0.988	1.089	0.667,1.778	0.73
Birth height (cm)	0.140	-0.447,0.727	0.639	0.256	-0.403,0.915	0.45
Birth weight						
Normal birthweight(2500-4000g)	1.106	0.511,2.395	0.798	0.94	0.428,2.066	0.88
Very low birth weight (<1500 g)	3.793	0.235,61.285	0.348	1.378	0.477,3.986	0.554
Low birth weight (<2500 g)	1.269	0.448,3.600	0.654	9.983	0.307,325.104	0.195
Fetal macrosomia (4000 g)	0.520	0.151,1.786	0.299	0.622	0.178,2.166	0.455

Analyses were adjusted for Gn dose, sperm concentration, normal fertilization rate, number of excellent embryos, fertilization method, ovarian stimulation protocols and infertility factor. OR, Odds ratio; CI, confidence interval.

### 3.5 Subgroup analysis

Given the significant heterogeneity in symptoms, infection chronology, and partner infection status among COVID-19-affected participants, we conducted stratified analyses to elucidate key factors influencing the impact of COVID-19 on pregnancy outcomes.

#### 3.5.1 Pregnancy outcomes by time from infection to oocyte retrieval

To evaluate the effect of the time interval between COVID-19 infection and oocyte retrieval on oocyte quality and adverse pregnancy outcomes, patients with COVID-19 infection were stratified into two subgroups based on the interval between infection and oocyte retrieval: recent infection group (defined as ≤ 28 days prior to retrieval) and remote infection group (28–84 days prior to retrieval). As is shown (**Table 4**), except for the non-infection group with 762 people, there are 65 patients in recent infection group, and 198 in remote infection group. Univariate and multivariate logistic regression analyses were conducted to assess the impact of infection timing on pregnancy outcomes (**Table 4**). After adjusting for potential confounders, the remote infection subgroup exhibited a significantly lower LBR (adjusted OR: 0.632, 95% CI: 0.450–0.887, *P* = 0.008 < 0.025) compared with the non-infection group following Bonferroni’s correction. No significant differences were observed in implantation rates, clinical pregnancy rates, biochemical pregnancy rates, or premature birth rates between the recent infection group and remote infection group.

**Table 4:** Univariate and multivariate logistic regression analysis of pregnancy outcomes by time interval between COVID-19 infection and oocyte retrieval.

Pregnancy outcomes	Non-infection group	≤28 day to retrieve	*P*1-value	28–84 day to retrieve	*P*2-value
Cycles, n	762	65		198	
Implantation, n (%)	602 (55.4)	63 (63)		172 (60.1)	
Crude OR (95% CI)	Ref	0.730 (0.478,1.115)	0.146	0.824 (0.632,1.075)	0.154
Adjusted OR (95% CI)	Ref	0.783 (0.508,1.209)	0.27	0.818 (0.620,1.079)	0.154
Clinical pregnancy, n (%)	428 (56.2)	34 (52.3)		101 (51.0)	
Crude OR (95% CI)	Ref	0.64 (0.262,1.567)	0.329	0.672 (0.381,1.186)	0.17
Adjusted OR (95% CI)	Ref	0.672 (0.266,1.696)	0.4	0.684 (0.378,1.237)	0.209
Biochemical pregnancy, n (%)	45(5.9)	4 (6.2)		12 (6.1)	
Crude OR (95% CI)	Ref	1.244 (0.413,3.751)	0.698	0.688 (0.299,1.584)	0.379
Adjusted OR (95% CI)	Ref	1.099 (0.352,3.432)	0.871	0.707 (0.299,1.674)	0.431
Early miscarriage, n (%)	22(5.5)	3 (10.7)		8 (8.1)	
Crude OR (95% CI)	Ref	2.056 (0.576,7.340)	0.267	1.506 (0.650,3.493)	0.34
Adjusted OR (95% CI)	Ref	2.118 (0.568,7.901)	0.264	1.418 (0.589,3.414)	0.436
LBR, n (%)	357(46.9)	25 (38.5)		70 (35.4)	
Crude OR (95% CI)	Ref	0.709 (0.422,1.192)	0.195	0.620 (0.449,0.858)	0.004*
Adjusted OR (95% CI)	Ref	0.727 (0.428,1.234)	0.237	0.632 (0.450,0.887)	0.008*
Premature birth, n (%)	22 (7.1)	1 (4.8)		4 (6.5)	
Crude OR (95% CI)	Ref	0.659 (0.084,5.143)	0.691	0.909 (0.302,2.737)	0.865
Adjusted OR (95% CI)	Ref	0.696 (0.086,5.617)	0.734	0.962 (0.314,2.949)	0.946

OR, Odds ratio; CI, confidence interval. Analyses were adjusted for Gn dose, sperm concentration, normal fertilization.

rate, ovarian stimulation protocols and infertility factor.

Pairwise comparisons between groups were adjusted by the Bonferroni method for multiple testing, with the adjusted.

significance level (α) calculated as 0.05/2 = 0.025. *Statistically significant with *P* < 0.025, *P*1: ≤28 day to.

retrieve group versus the non-infection group; *P*2: 28–84 day to retrieve group versus the non-infection group.

#### 3.5.2 Association between fever severity and LBR in the remote infection group

COVID-19 exhibits a heterogeneous clinical spectrum, ranging from subclinical infection to critical systemic inflammation. Fever severity, a quantifiable and clinical marker of systemic inflammatory response, serves as a measurable indicator for infection intensity. We then further evaluated the impact of fever severity on LBR in remote infection group. During the patient’s visit, the patient’s body temperature is measured by the nurse using an infrared thermometer and recorded. As the study found that body temperature ≥ 38.5°C significantly impairs follicular development during ovulation induction cycles (22), patients were stratified into moderate-to-low fever (< 37.2°C and < 38.5°C) and high fever (≥ 38.5°C) subgroups. Among the 198 patients in remote infection group, 102 (51.5%) manifested moderate-to-low fever (< 37.2°C and < 38.5°C), whereas 96 (48.5%) developed high fever (≥ 38.5°C) (**Table 5**). After adjusting for potential confounders multivariate logistic regression analysis demonstrated a significantly reduced LBR in the high-fever subgroup compared to the non-infected reference group (adjusted OR: 0.467, 95% CI: 0.290–0.752, *P* = 0.002 <0.025) following Bonferroni’s correction (**Table 5**). The remote infection with a high fever of ≥ 38.5°C subgroup had a more reduced LBR and an elevated mid-to-late miscarriage rate relative to the non-infected group. These data indicate that higher fever (≥ 38.5°C) during COVID-19 adversely affect reproductive outcomes, particularly within the critical 28 to 84 day window post-infection.

**Table 5:** Univariate and multivariate logistic regression analyses of LBR by fever magnitude in the non-infection and remote infection groups.

Groups	LBR, n (%)	Crude OR (CI)	*P*1-value	Adjusted OR (CI)	*P*2-value
Non-infection group (n = 762) (< 37.2°C)	357 (46.9)	Ref		Ref	
remote infection group (n = 198) (28–84 days prior to retrieval)					
< 37.2°C and < 38.5°C (n = 102)	42 (41.2)	0.794 (0.522,1.208)	0.281	0.792 (0.513,1.223)	0.292
≥ 38.5°C (n = 96)	28 (29.2)	0.467 (0.294,0.742)	0.001	0.467 (0.290,0.752)	0.002*

OR, Odds ratio; CI, confidence interval. Analyses were adjusted for Gn dose, sperm concentration, normal fertilization rate, ovarian stimulation protocols, infertility factor.

Pairwise comparisons between groups were adjusted by the Bonferroni method for multiple testing, with the adjusted significance level (α) calculated as 0.05/2 = 0.025. *Statistically significant with *P* < 0.025, *P*1: < 38.5°C group versus the non-infection group; *P*2: ≥ 38.5°C group versus the non-infection group.

#### 3.5.3 Pregnancy outcomes in non-infection, female-only infection or dual-partner infection groups

To evaluate whether dyadic COVID-19 infection (concurrent infection in both partners) potentiates additional reproductive risks beyond individual exposures, we compared pregnancy outcomes between the two groups. As is showed, 63 patients were female-only infection, while 200 was dual-partner infection (**Table 6**). Multivariate logistic regression models, adjusted for potential confounders, revealed that the dual-partner infection group exhibited a significantly lower LBR (LBR; adjusted OR = 0.591, 95% CI: 0.421–0.830, *P* = 0.002 < 0.025) and a higher mid-to-late miscarriage rate (adjusted OR = 7.871, 95% CI: 2.473–25.057, *P* < 0.001) compared to the female-only infection group following Bonferroni’s correction (**Table 6**). No statistically significant differences were observed in implantation rate, clinical pregnancy rate, early miscarriage rate, ectopic pregnancy rate, or preterm birth rate after adjustment. These findings suggest that dual-partner infection may compound adverse pregnancy outcomes, particularly impacting late gestational stability and live birth potential.

**Table 6:** Univariate and multivariate logistic regression analysis of pregnancy outcomes in non-infection, female-only infection or dual-partner infection groups.

Neonatal outcomes	Non-infection group	Female-only infection group	*P*1-value	Dual-partner infection group	*P*2-value
Cycles, n	762	63		200	
Implantation, n (%)	602 (55.4)	56 (57.1)		179 (62.2)	
Crude OR (95% CI)	Ref	0.933 (0.614,1.416)	0.744	0.757 (0.580,0.989)	0.041
Adjusted OR (95% CI)	Ref	0.976 (0.636,1.498)	0.912	0.757 (0.573,1.000)	0.050
Clinical pregnancy, n (%)	428 (56.2)	38 (60.3)		97 (48.5)	
Crude OR (95% CI)	Ref	0.701 (0.289,1.701)	0.432	0.651 (0.368,1.150)	0.139
Adjusted OR (95% CI)	Ref	0.736 (0.296,1.832)	0.51	0.662 (0.365,1.198)	0.173
Biochemical pregnancy, n (%)	45 (5.9)				
Crude OR (95% CI)	Ref	1.149 (0.383,3.443)	0.804	0.706 (0.306,1.628)	0.414
Adjusted OR (95% CI)	Ref	1.141 (0.367,3.541)	0.82	0.696 (0.294,1.649)	0.41
Early miscarriage, n (%)	22 (5.5)	2 (5.7)		9 (9.8)	
Crude OR/β (95% CI)	Ref	1.039 (0.234,4.611)	0.96	1.858 (0.826,4.182)	0.134
Adjusted OR/β (95% CI)	Ref	1.638 (0.306,8.760)	0.564	0.634 (0.130,3.091)	0.573
Mid to late miscarriage, n (%)	6 (1.5)	3 (8.6)		9 (9.8)	
Crude OR (95% CI)	Ref	6.141 (1.467,25.711)	0.013	7.102 (2.461,20.496)	<0.001*
Adjusted OR (95% CI)	Ref	8.101 (1.719,38.175)	0.008	7.871 (2.473,25.057)	<0.001*
Ectopic pregnancy, n (%)	12 (3)	2 (5.7)		2 (2.2)	
Crude OR (95% CI)	Ref	1.955 (0.420,9.104)	0.393	0.717 (0.158,3.259)	0.666
Adjusted OR (95% CI)	Ref	1.638 (0.306,8.760)	0.564	0.634 (0.130,3.091)	0.573
LBR, n (%)	357 (46.9)	27 (42.9)		68 (34.0)	
Crude OR (95% CI)	Ref	0.851 (0.506,1.430)	0.542	0.584 (0.422,0.809)	0.001*
Adjusted OR (95% CI)	Ref	0.891 (0.524,1.514)	0.67	0.591 (0.421,0.830)	0.002*
Premature birth, n (%)	22 (7.1)	3 (12.5)		2 (3.4)	
Crude OR (95% CI)	Ref	1.883 (0.521,6.807)	0.334	0.463 (0.106,2.022)	0.306
Adjusted OR (95% CI)	Ref	2.082 (0.546,7.945)	0.283	0.478 (0.107,2.139)	0.334

Analyses were adjusted for Gn dose, sperm concentration, normal fertilization rate, ovarian stimulation protocols, infertility factor.

OR, Odds ratio; CI, confidence interval.

Pairwise comparisons between groups were adjusted by the Bonferroni method for multiple testing, with the adjusted significance level (α) calculated as 0.05/2 = 0.025. *Statistically significant with *P* < 0.025; *P*1: Only the female infected group versus the non-infection group; *P*2: Both partners infected group versus.

the non-infection group.

## 4 Discussion

In this study, we have made several important observations.

Firstly, we found COVID-19 infection did not affect oocyte retrieval counts, embryo implantation rates, and clinical or biochemical pregnancy rates. But COVID-19 infection significantly reduces LBR and increases mid-to-late miscarriage rates in fET cycles. Similarly, previous studies also reported that COVID-19 infection does not significantly impact embryo implantation, clinical pregnancy, ongoing pregnancy or preterm birth rates (14, 23). Existing research has mainly focused on COVID-19’s impact on early pregnancy IVF outcomes, with limited attention to its effects on LBR and neonatal outcomes. Notably, acting as a cellular entry target of SARS-CoV-2 virus, ACE2 is highly expressed in oocytes, follicles, blastocysts (7, 23), and human placenta during pregnancy (9). These suggested that COVID-19 infection may affect the LBR and neonatal outcomes of IVF. After adjusting for potential confounders, we further confirmed that COVID-19 infection significantly reduced LBR and increased mid-to-late miscarriage rates. Similarly, Violet Eckstein et al. reported that there was a significantly higher miscarriage rate at 34.6% in 2022, compared to 19.7% in the pre-pandemic years of 2018–2019 (16). However, another study suggested that embryo development, pregnancy and live birth outcomes following frozen embryo transfer cycles were not compromised during the COVID−19 pandemic (24). The inconsistency between their results and our data could be attributed to their limited sample size and different embryo transfer method. These findings raised concerns about the potential impact of COVID-19 infection during ART treatment on LBR.

Secondly, we found that the time interval between COVID-19 infection and oocyte retrieval, infection severity and dual infection are risk factors that adversely affect the birth and post-birth outcomes of IVF. ART involves multiple stages, including ovulation induction, *in vitro* fertilization, fET. fET following COVID-19 infection presents a series of complex challenges, including abnormal immune responses post-infection and imbalanced hormone levels induced by ovulation stimulation. Consequently, investigating the specific factor that impact the effect of COVID-19 infection on fET outcomes is of particular significance. Further stratification of the infected cohort into recent and remote infection subgroups, we demonstrated that remote infection leads to a significantly lower LBR. The remote infection with a high fever of ≥ 38.5°C subgroup had a markedly reduced LBR relative to the non-infection group. Additionally, dual-partner COVID-19 infections were also associated with a decreased LBR and an elevated mid-to-late miscarriage rate. Similarly, COVID-19 infection more than 180 days prior to retrieval 44 had a negative effect on oocyte yield (14). COVID-9 infection during controlled ovarian stimulation (COS) has been linked to reduced embryo and blastocyst quality (25, 26). Li et al. reported that individuals infected COVID-19 during IVF treatment may experience a lower percentage of top-quality embryos (27), reduced clinical pregnancy rates, and increased preterm birth incidence (28, 29). Research indicates that ovarian tissue scores decrease following heat stress, suggesting that high temperatures may cause pathological damage to the ovaries (30). Emerging evidence suggests that long COVID-9 involves dysregulated immune responses, such as significant alterations in myeloid and lymphocyte populations and exaggerated anti-SARS-CoV-2 humoral immunity in COVID-9 patients (2, 31). The immunological and endocrinal disturbances induced by COVID-9 infection may contribute to the observed reduction in LBR and increased mid-to-late pregnancy loss in infected patients undergoing fET.

This study has several limitations. Firstly, the single-center and homogeneous cohort design may restrict the generalizability of the findings Secondly, further research should investigate whether long COVID-19 directly contributes to adverse pregnancy outcomes following fET, particularly in cases involving severe illness or dual-partner infection.

## 5 Conclusion

In conclusion, our findings suggested that COVID-19—particularly post-acute phase infections (28–84 days) with high-grade fever (≥ 38.5°C) and couple-concordant transmission—compromises reproductive success in fresh IVF cycles, as evidenced by reduced LBR. Interestingly, these adverse effects appear limited to maternal outcomes, with neonatal outcomes remaining comparable between groups. These results highlight the importance of developing evidence-based clinical protocols to optimize the timing of fertility treatments following COVID-19 infection, with consideration for patients experiencing febrile illness or when both partners have been infected. Future studies should be expanded to multi-center settings and enroll a substantially larger cohort of infected women or couples to validate this finding.

## Data Availability

The original contributions presented in the study are included in the article/**Supplementary Material**. Further inquiries can be directed to the corresponding authors.
