# Supplementary material for: The impact of COVID-19 infection on the live birth rate in fresh embryo transfer cycles
Source: Front Endocrinol (Lausanne). 2026 Apr 17;17:1764229. doi: 10.3389/fendo.2026.1764229 (PMC13132749; doi:10.3389/fendo.2026.1764229)
Supplement: Supplementary file 1 [file Table1.docx]

| **Supplementary table 1 Clinical outcomes in the two groups.** | | |  |
| --- | --- | --- | --- |
|  | **Non-infection group** | **Infection group** | ***P*-value** |
| OHSS rate, n (%) | 9 (1.2) | 2 (0.8) | 0.565 |
| Implantation rate, n (%) | 484 (44.6) | 151 (39.1) | 0.063 |
| Clinical pregnancy rate, n (%) | 428 (56.2) | 135 (51.33) | 0.174 |
| Biochemical pregnancy rate, n (%) | 45 (5.9) | 16 (6.1) | 0.916 |
| Early miscarriage rate, n (%) | 22 (5.5) | 11 (8.7) | 0.203 |
| Mid to late miscarriage Rate, n (%) | 6 (1.5) | 12 (9.4) | <0.001* |
| Ectopic pregnancy rate, n (%) | 12 (3.0) | 4 (3.1) | 0.935 |
| LBR, n (%) | 357 (46.9) | 95 (36.1) | 0.003* |
| Premature birth rate, n (%) | 22 (7.1) | 5 (6.0) | 0.742 |
| Notes: Categorical variable is presented as n (%).  *Statistically significant with *P* < 0.05. | | |  |

**Supplementary table 2 Neonatal outcomes in the two groups.**

|  | **Non-infection group** | **Infection group** | ***P*-value** |
| --- | --- | --- | --- |
| Number of newborns, n | 402 | 107 |  |
| Gender of newborn, n (%) |  |  | 0.988 |
| Male | 222 (55.2) | 59 (55.1) |  |
| Female | 180 (44.8) | 48 (44.8) |  |
| Birth height (cm), median (Q1, Q3) | 50 (50, 51) | 49.62 ± 2.79 | 0.621 |
| Number of singletons, (n) | 312 | 84 |  |
| Very low birth weight, n (%) | 1 (0.3) | 2 (1.2) | 0.377 |
| Low birth weight, n (%) | 15 (4.8) | 5 (6.0) | 0.584 |
| Fetal macrosomia, n (%) | 21 (6.7) | 3 (3.6) | 0.291 |

Notes: Data are shown as median (Q1, Q3) or number (percentage).
